# Transcription factors underlying wing margin color patterns and pupal cuticle markings in butterflies

**DOI:** 10.1186/s13227-020-00155-w

**Published:** 2020-05-27

**Authors:** Robert D. Reed, Jayne E. Selegue, Linlin Zhang, Craig R. Brunetti

**Affiliations:** 1 Department of Ecology and Evolutionary Biology, Cornell University, 215 Tower Road, Ithaca, NY 14853-7202 USA; 2 School of Pharmacy, University of Wisconsin, 777 Highland Ave, Madison, WI 53705 USA; 3 Institute of Oceanology, Chinese Academy of Sciences, 7 Nanhai Road, Qingdao, 266003 China; 4 Department of Biology, Trent University, 1600 East Bank Dr., Peterborough, ON K9J 7B8 Canada

**Keywords:** *Junonia coenia*, *Bicyclus anynana*, Butterfly, Wing patterning, Spalt, Distal-less, Eyespot, Marginal bands

## Abstract

**Background:**

The diversity of butterfly color patterns can be attributed to a relatively small number of pattern elements that are homologous across Lepidoptera. Although genes involved in patterning some of these elements have been identified, the development of several major elements remains poorly understood. To identify genes underlying wing pupal cuticle markings and wing margin color patterns, we examined expression of the candidate transcription factors Engrailed/Invected (En/Inv), Distal-less (Dll), Cubitus interruptus (Ci), and Spalt in two nymphalids: *Junonia coenia* and *Bicyclus anynana*.

**Results:**

We found that En/Inv, Dll, and Ci mark domains on the *J. coenia* last-instar forewing disc that closely correspond to the position and shape of pupal cuticle markings. We also found that Spalt demarcates wing margin color patterns in both *J. coenia* and *B. anynana*, and that CRISPR/Cas9 deletions in the *spalt* gene result in reduction and loss of wing margin color patterns in *J. coenia*. These data demonstrate a role for *spalt* in promoting wing margin color patterning, in addition to its previously described role in eyespot patterning.

**Conclusion:**

Our observations support the model that a core set of regulatory genes are redeployed multiple times, and in multiple roles, during butterfly wing pattern development. Of these genes, *spalt* is of special interest as it plays a dual role in both eyespot and margin color pattern development.

## Background

Butterflies are distinguished by the diverse color patterns they bear upon their wings. As originally proposed by Schwanwitsch [1] and Süffert [2], and later refined by Nijhout [3], this spectacular diversity of color patterns is the product of a relatively simple ground plan of evolutionarily conserved pattern elements that are homologous across Lepidoptera. These elements, many of which are characterized as symmetry systems, include the marginal and submarginal bands, the border ocelli, the central symmetry system, the basal symmetry system, and the wing root band [3]. Lepidopteran wing pattern diversity is thought to be largely derived by gains, losses, and permutations of these various pattern elements. Of these core ground plan elements, the border ocelli system, which gives rise to eyespot color patterns in nymphalid butterflies, is one of the best studied.

The border ocelli system runs parallel to the wing margin of butterflies and typically consists of a row of eyespots, and in some taxa, additional merged patterns proposed to be derived from eyespots [4]. Eyespot patterns themselves are usually composed of concentric rings of colored scales and can play a role in predator avoidance [5] as well as mate selection [6] where females focus on the eyespot brightness and presence of the UV-central scales in males [7]. Eyespot formation is promoted by a group of signaling cells, at the center of the eyespot, known as the focus [8, 9]. When focal cells are transplanted to different regions of the butterfly wing during early pupal development, the original eyespot is lost, and an ectopic eyespot pattern is formed [8–10], suggesting that focal cells are necessary and sufficient for organizing the eyespot. The most likely explanation for these observations is that the focal cells are either secreting a diffusible morphogen or acting as a sink for a morphogen [10]. Cells surrounding the focus would therefore differentiate into colored scales based on their distance from the focus, due to positional differences in morphogen concentration.

Determination and elaboration of eyespots has been proposed to occur in four stages based on transplantation, ablation, and gene expression studies [8, 11]. During the first stage, in the imaginal discs of mid-stage last-instar larvae, prepattern expression of regulatory factors, including Spalt, Notch (N), and Dll, mark potential and actual locations of eyespot foci [8, 12–15]. During the second stage, in late last-instar wing discs, these sometimes-transient prepatterns resolve into distinct expression domains that precisely predict the locations of adult eyespot color patterns. At this time a number of signaling molecules and transcription factors mark presumptive eyespot foci, including Dll, En/Inv, Patched (Ptc), Hedgehog (Hh), Ci, N, Wnt ligands, and Spalt [8, 12–14, 16, 17]. The third stage occurs in early pupae, when signaling from the focus induces surrounding cells to produce elaborated patterns of concentric gene expression domains that presage the final adult color patterns. Although the specific molecular identity of the focal signal has not been demonstrated experimentally, transcripts of the signaling ligand Wingless (Wg), WntA, and a member of the TGF-β pathway are expressed in eyespot foci shortly after pupation [14, 18], and wingless RNAi knockdowns can cause eyespot size reduction [19]. In the fourth and final stage, the cells organized by the focus differentiate into scale-building cells that ultimately produce the colors seen on the adult wing. The transcription factors En/Inv, Spalt, and Dll are proposed to play a role in defining different color-specific populations of scale-building cells in response to the focal signal [20], which is interesting because these same genes also appear to play an earlier, and very different, role in focal determination, as described above.

This previous work on eyespot development has highlighted several candidate genes involved in the development of eyespots and the border ocelli system. There is still much we do not understand, however, especially in terms of gene function and interaction. One observation of interest is that many eyespot-associated genes also show expression correlated with various other non-eyespot patterns, thus implying co-option from, and/or developmental integration between, different patterning systems. Most obviously En/Inv, Ptc, Hh, and Ci appear to retain their ancestral roles in anterior–posterior compartmentalization in addition to derived roles in focal determination. Also of interest are the complex and temporally dynamic patterns of Wg, WntA, N, Dll, and Spalt expression, which suggest that these genes may function in the development of multiple color pattern elements. Most notably, all of these genes show expression along the wing margin [12, 13, 18, 21], in addition to their better characterized roles in symmetry system and/or eyespot development. Supporting this is recent CRISPR/Cas9 mosaic knockout experiments that show Spalt and Dll influence the development of both eyespot and non-eyespot wing patterns [17, 21]. Furthermore, some of these genes show expression patterns that could potentially be correlated with pupal cuticle patterns. Indeed, Taira and Otaki [22] recently suggested that the eyespot focus may also be mediating the formation of pupal cuticle spots on the wings of *Junonia* butterflies.

Here we provide a more detailed characterization of non-eyespot color pattern-related expression of the presumptive border ocelli system factors En/Inv, Dll, Ci, and Spalt in both forewing and hindwing last-instar imaginal discs and pupal wings from *Junonia coenia* and *Bicyclus anynana*. We found that some of these proteins show expression patterns that imply roles in defining pupal cuticle spots. We also observe strong associations between Spalt expression and wing margin color patterns. Consistent with this, we report CRISPR/Cas9 mosaic knockouts of *spalt* that show a related loss of submarginal band color patterns.

## Results

### Dll, En/Inv, and Ci expression demarcate pupal cuticle markings

**Fig. 1:**
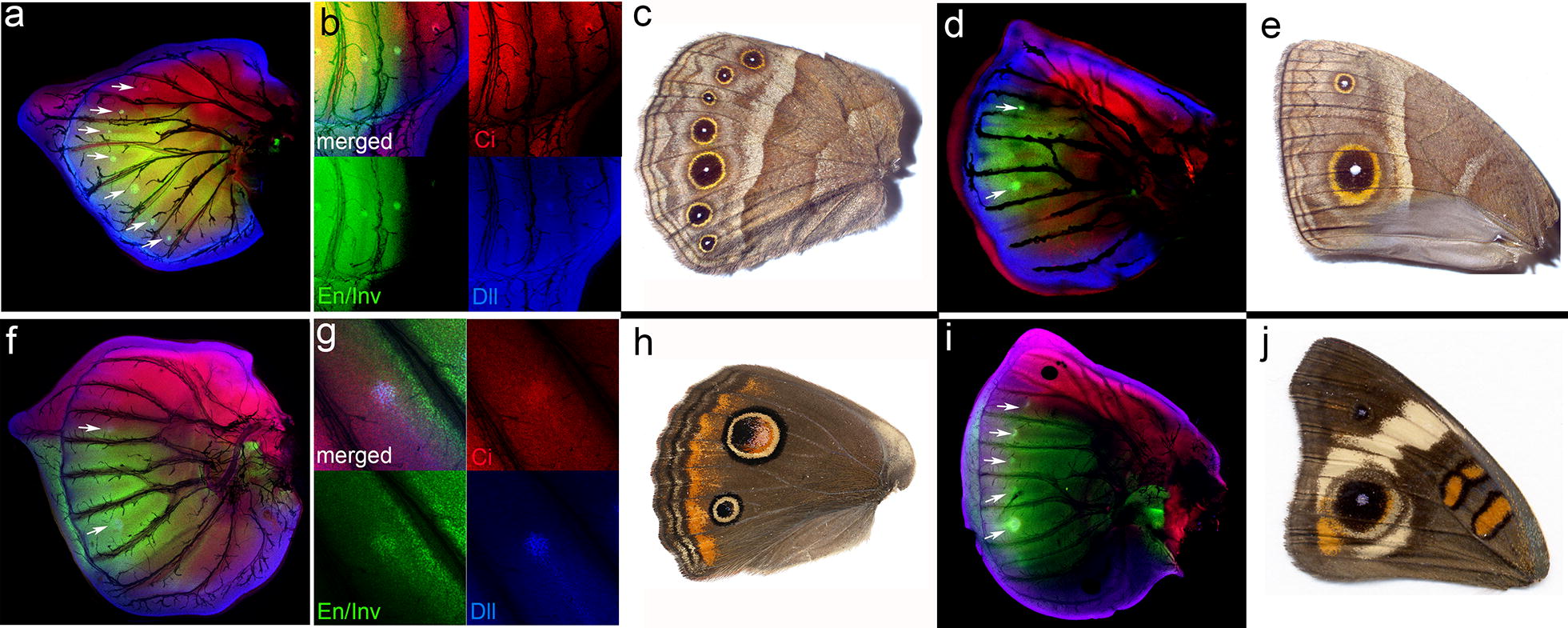
Expression of patterning proteins in last-instar larval wing discs of *J. coenia* and *B. anynana. B. anynana* hindwing (**a**, **b**) and forewing (**d**) and *J. coenia* hindwing (**f**, **g**) or forewing (**i**) last-instar wing imaginal discs were excised and indirect immunofluorescence was performed to detect for the presence of Engrailed/Invected (green), distal-less (blue), and cubitus interruptus (red). The *B. anynana* (**b**) and *J. coenia* (**g**) hindwing staining showing the individual protein staining patterns along with the merged image. The corresponding adult hindwings of *B. anynana* (**c**) and *J. coenia* (**h**) and adult forewings of *B. anynana* (**e**) and *J. coenia* (**j**) butterflies are also shown. White arrows highlight the eyespot foci

Immunostaining revealed distinct expression of En/Inv, Dll, and Ci marking the position of adult eyespot foci in last-instar *B. anynana* forewing and hindwing imaginal discs (Fig. 1a–e), as previously reported [8, 14, 16]. Similarly, there was a correlation between last-instar hindwing disc En/Inv, Dll, and Ci expression and eyespot foci in *J. coenia* (Fig. 1f–h). In *J. coenia* forewings, however, where there are only two adult eyespots (Fig. 1j), we observed five spots of En/Inv, Dll, and Ci expression in late last-instar forewing discs (Fig. 1i, white arrows), a phenomenon which had previously been reported [23]. It is notable that En/Inv, Dll, and Ci, which have been implicated in focal determination, are found in regions of the forewing that do not give rise to actual eyespot color patterns (Fig. 1i, j).

**Fig. 2:**
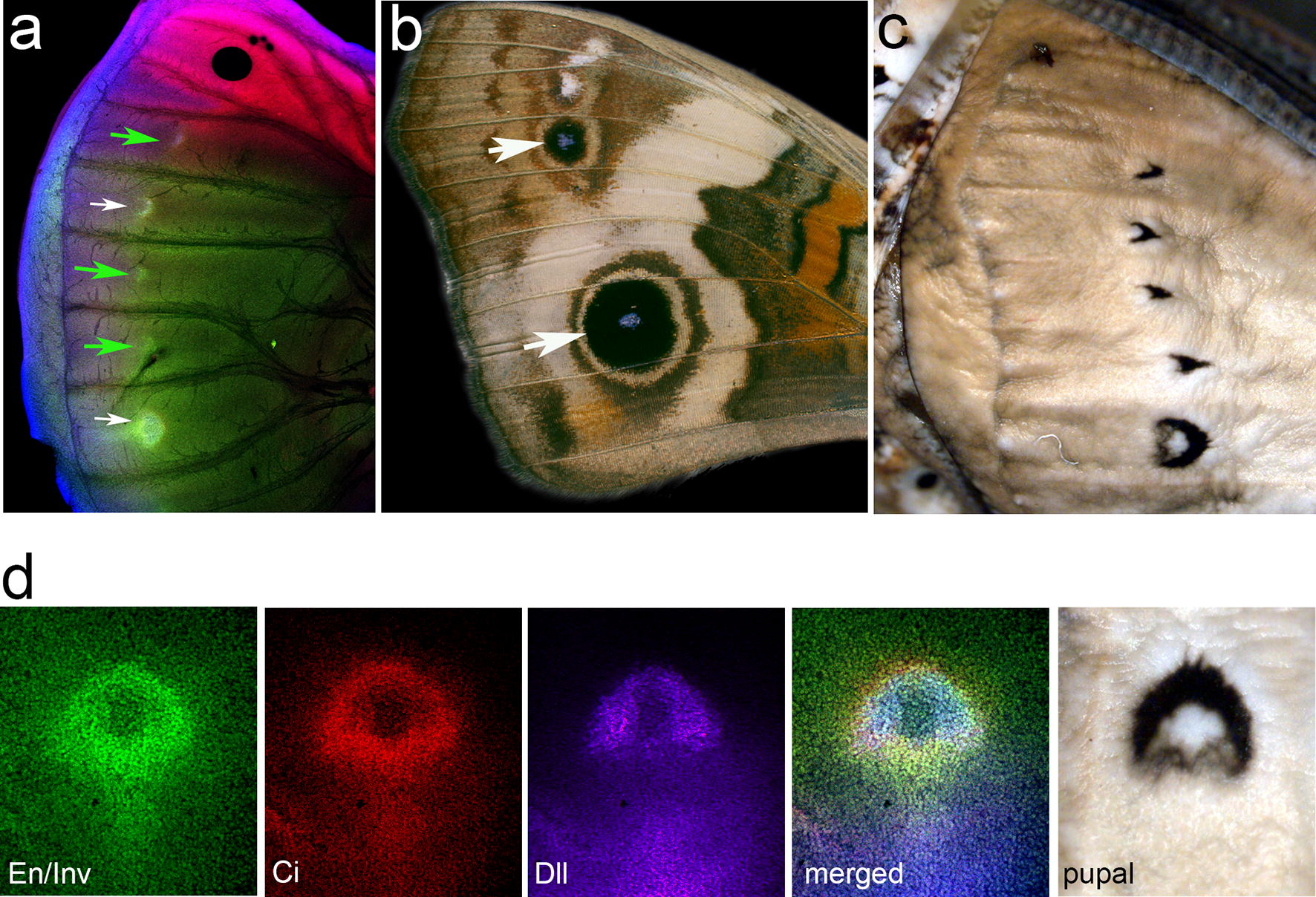
Protein expression predicts wing and pupal case color patterns. **a** Immunofluorescent detection of proteins Dll (blue), En/Inv (green), and Ci (red) in last-instar *J. coenia* forewing imaginal discs. The white arrows highlight expression predictive of future eyespot foci, while the green arrows highlight the foci that do not correspond to adult eyespots. **b** An adult *J. coenia* forewing with white arrows highlighting the location of eyespot foci predicted by gene expression in **a**. **c***J. coenia* forewing pupal cuticle. **d** The large eyespot focus in the last-instar *J. coenia* forewing disc was examined using indirect immunofluorescence for the proteins Dll (blue), En/Inv (green), and Ci (red) along with the corresponding region of the *J. coenia* pupal cuticle

Since both *B. anynana* and *J. coenia* each have two eyespots on the adult forewing in the exact same position on the wing, we wondered why *J. coenia* last-instar larva exhibited additional spots of En/Inv, Dll, and Ci expression. Closer examination of *J. coenia* immunostains revealed that the expression patterns (Fig. 2a) are not consistent with typical round eyespot foci (Fig. 2b), rather they appear as chevrons and circles (Fig. 2a). These staining patterns are strongly correlated with the position and shape of the black markings on the pupal wing cuticle (Fig. 2c). This is most strikingly evident when comparing the large circular spot on the pupal cuticle with late last-instar staining patterns (Fig. 2d).

### Spalt defines eyespot and non-eyespot pattern elements in the border ocelli system

Previous studies have shown that Spalt is expressed in the last-instar wing discs of *B. anynana* and *J. coenia* at the presumptive sites of eyespot focus formation [14, 23]. Interestingly, Spalt is also expressed in territories of the pupal wing that correspond to black patches on the adult *Pieris rapae* wing, independent of the eyespot developmental program [14, 24], suggesting that this transcription factor may function in patterning elements other than the border ocelli system. To further explore the role of Spalt during eyespot and wing patterning, we more closely examined its expression in larval and pupal wings of *J. coenia* and *B. anynana*.

**Fig. 3:**
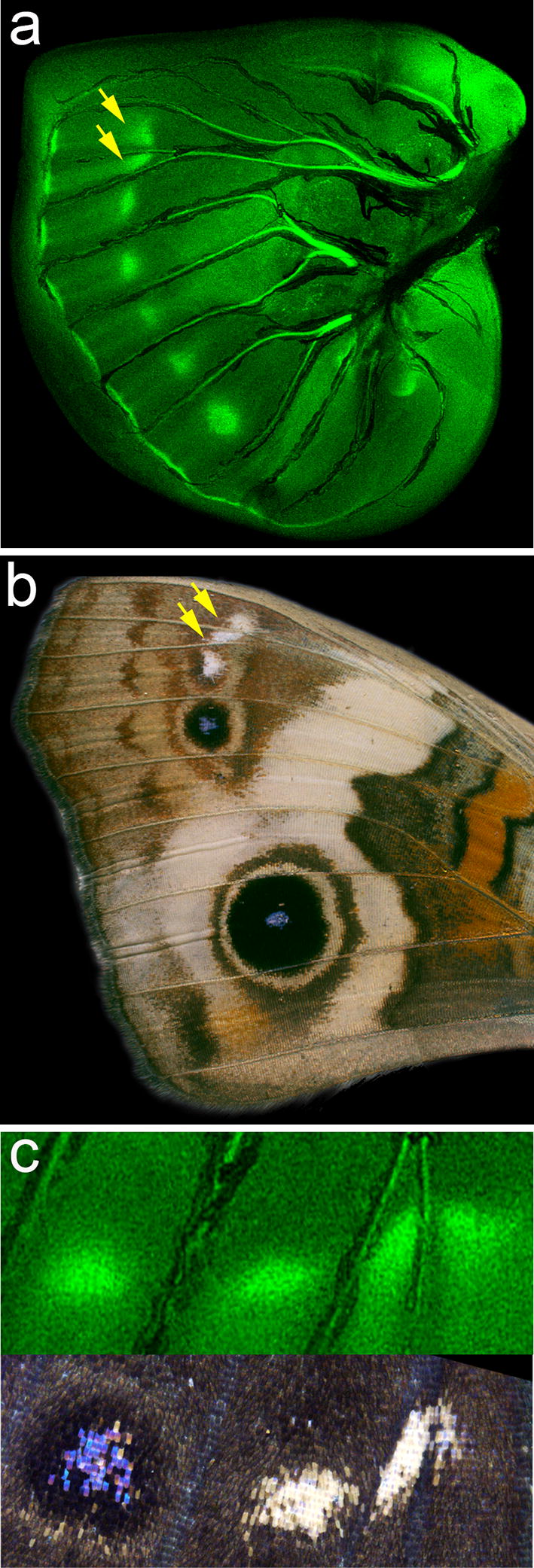
*spalt* predicts non-eyespot elements of the border ocelli pattern system. **a** Immunofluorescent detection of Spalt (green) in late last-instar *J. coenia* forewing. **b** An adult *J. coenia* forewing. Yellow arrows highlight white anterior spots associated with Spalt expression. **c** Comparison of adult color *J. coenia* anterior forewing color patterns with Spalt (green) expression

While En/Inv, Dll, and Ci expression marks five spots on the forewing disc of *J. coenia* (Fig. 2a), Spalt expression occurs in an additional two anterior spots, for a total of seven spots (previously reported in [23]) (Fig. 3a). If we presume five of the Spalt spots contribute to the two *J. coenia* eyespots seen on the adult wing, and possibly with the five black pupal cuticle markings, then we are left with the question of any potential roles for the two anterior-most Spalt spots (Fig. 3a, yellow arrows). Examination of the *J. coenia* adult forewing reveals two white marks (Fig. 3b, yellow arrows) that are precisely predicted by these anterior Spalt expression domains (Fig. 3c). The close correspondence of expression leads us to speculate that Spalt may play a role in determining these color pattern elements, and by extension, that they may be elements of the border ocelli system.

### Spalt defines submarginal bands

**Fig. 4:**
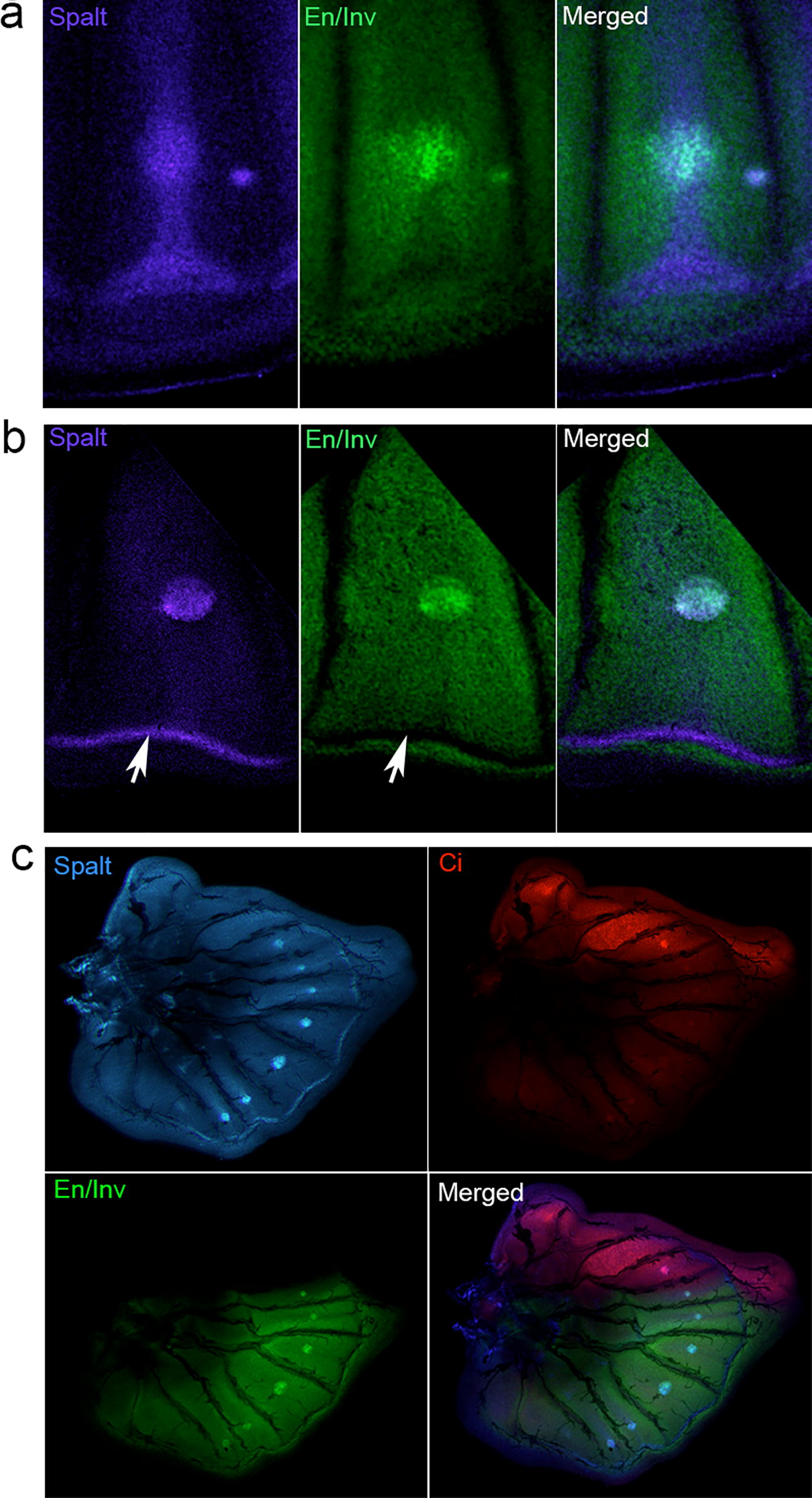
Spalt expression in eyespot foci and future wing margins of *J. coenia* and *B. anynana.* Immunofluorescent detection of Spalt in **a** early and **b** late last-instar *J. coenia* forewings, for En/Inv (green) and Spalt (blue). **c** Immunofluorescent detection of Spalt in late last-instar *B. anynana* hindwing discs, for En/Inv (green), Ci (red), and Spalt (blue)

In addition to the border ocelli system expression, we also observed distinct Spalt expression along the border lacuna (i.e., the apoptosis boundary that will become the adult wing margin) [25] of last-instar imaginal discs (Figs. 3a, 4). In *J. coenia*, Spalt expression was observed along the wing margin that resolved into a sharp band running along the proximal edge of the border lacuna (Figs. 3a, 4a, b). Interestingly, En/Inv and Spalt expression do not overlap, as the marginal stripe of Spalt is an area devoid of En/Inv expression (Fig. 4b, white arrow). In last-instar hindwing discs of *B. anynana*, we observed similar wing margin Spalt expression, in addition to expression in the eyespot foci (Fig. 4c).

**Fig. 5:**
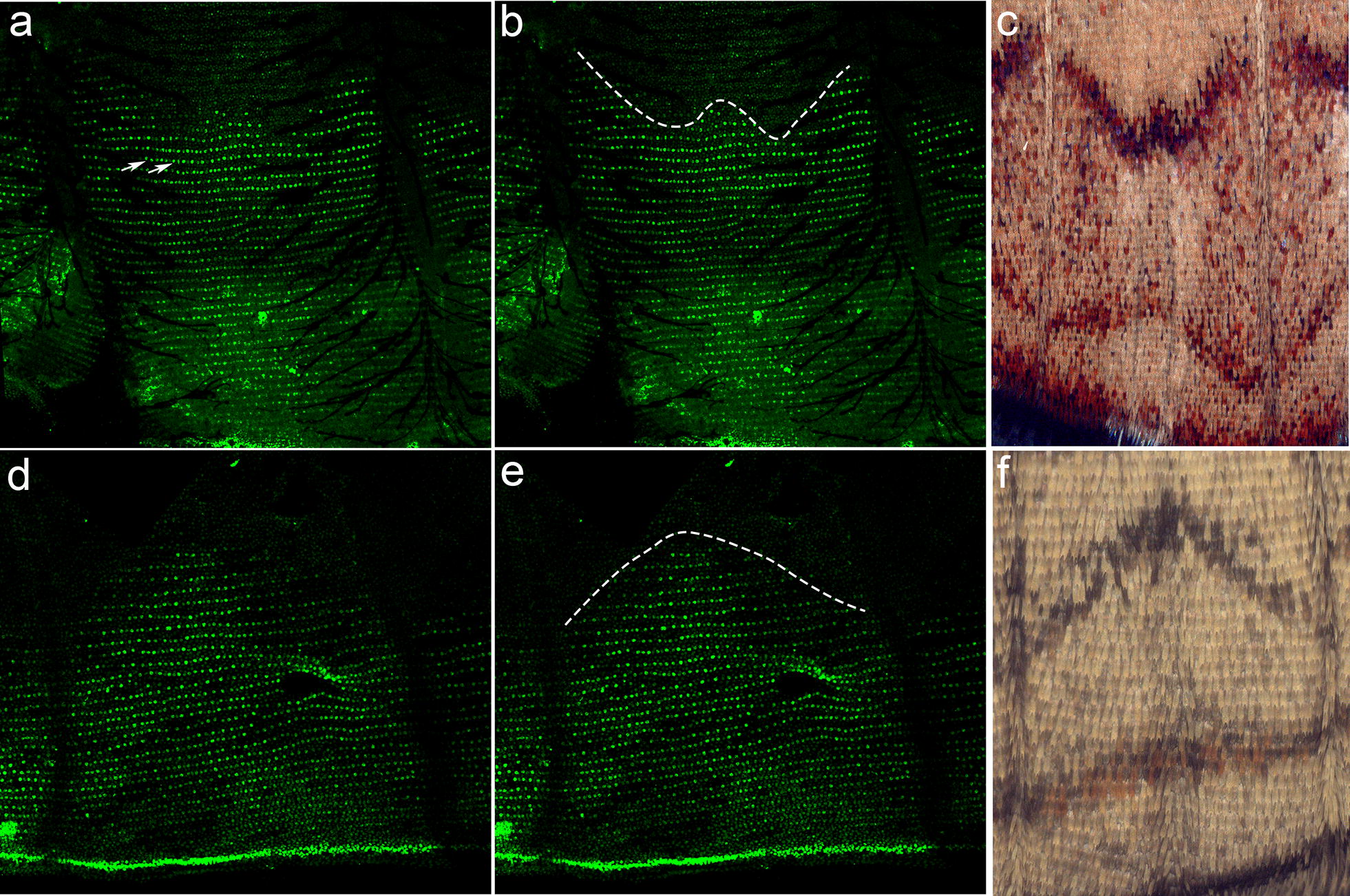
Spalt defines wing margin color pattern boundaries in *J. coenia* and *B anynana*. Immunofluorescent detection of Spalt (green) in 16–24 h pupal wings of *J. coenia* (**a**, **b**) and *B. anynana* (**d**, **e**). The dashed lines in **b** and **e** illustrated the boundaries of Spalt-positive versus Spalt-negative cells that correspond to wing color pattern boundaries in *J. coenia* (**c**) and *B. anynana* (**f**)

To more thoroughly explore the role Spalt may play in defining wing margin color patterns, we examined its expression during pupal development, at the post-signaling stage when eyespot color pattern expression is fully elaborated. In 16–24 h pupal forewings of both *J. coenia* (Fig. 5a–c) and *B anynana* (Fig. 5d–f) Spalt expression showed a remarkably precise association with wing margin color patterns. For example, the submarginal bands (Externa III, or EIII, sensu Schwanwitsch) of adult *J. coenia* are “W” shaped (Fig. 5c), and the interface between spalt-positive and spalt-negative scales at the wing margin shows a similar “W” pattern (Fig. 5a, b). Similarly, *B. anynana* submarginal bands display a chevron-like pattern (Fig. 5f) and the interface of Spalt-positive and Spalt-negative scales demarcates the same chevron-like pattern (Fig. 5d, e). These data suggest that Spalt may play a role in defining non-eyespot color patterns along with wing margin.

**Fig. 6:**
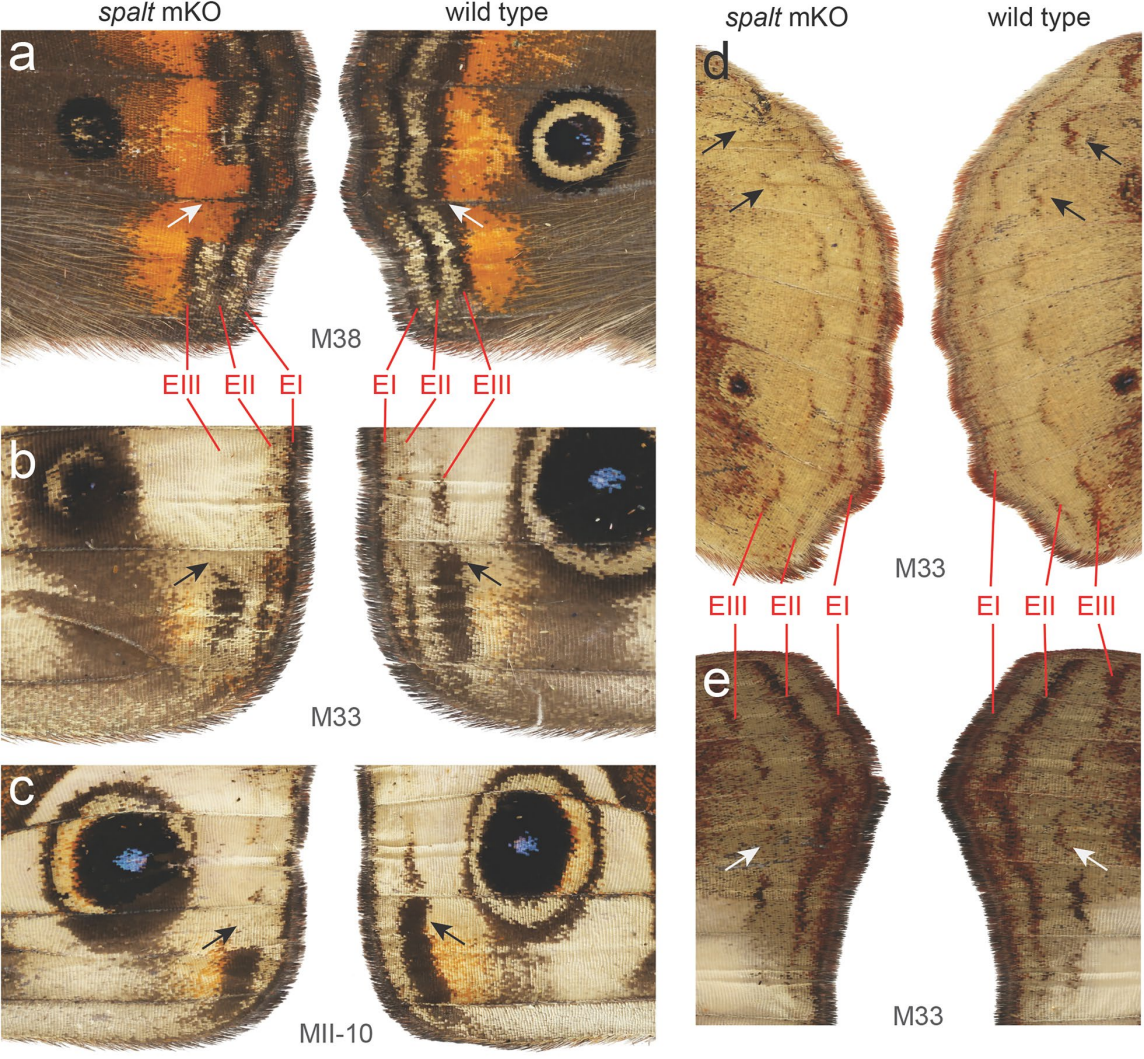
CRISPR/Cas9 mosaic *spalt* deletion knockouts results in loss of wing margin bands. Mosaic knockouts (mKOs) of *spalt* in *J. coenia*. Left and right wings are shown from the same animal to demonstrate asymmetric mosaic phenotypes. **a** Loss of EIII in dorsal hindwing. **b**, **c** Loss of EIII in ventral forewings. Loss of “W”-shaped EIII submarginal bands in **d** ventral hindwing and **e** ventral forewing

To functionally confirm a role for Spalt in defining the marginal band, we examined CRISPR/Cas9 *spalt* deletion mosaics in *J. coenia*, produced during a previous screen [21]. Mosaic deletions of Spalt resulted in disruption and loss of EIII submarginal band color patterns in both forewings and hindwings (Fig. 6a–e). This effect is in addition to previously described reduction-of-eyespot phenotypes [21], and indeed many of the mosaic butterflies that showed disruption of marginal bands also showed reduction and/or loss of eyespots (e.g., Fig. 6a–c). These results provide direct evidence, consistent with the immunostaining results, that Spalt plays a functional role in defining wing submarginal band color patterns in butterflies.

## Discussion

In this report, we demonstrate the evolutionary and developmental flexibility of the regulatory networks underlying butterfly wing patterning. Virtually all the wing patterning genes thus far identified in butterflies are known to play other deeply conserved, non-wing patterning roles in insect development. This has been recognized since the first gene expression patterns were reported in butterflies [12], and led to wing patterns serving as a popular illustrative case study of gene co-option. Here we undertook an expanded exploration for wing patterning functions for En/Inv, Dll, Ci, and Spalt. These transcription factors have all been proposed to have been co-opted to eyespot development from various ancestral functions, including appendage development, anterior–posterior compartmentalization, and wing vein development [26–29]. In this study we asked whether these genes may play some additional roles in wing patterning beyond eyespot development, and we presented new evidence for their likely roles in wing pupal cuticle marking and, in the case of Spalt, wing margin color pattern determination as well.

### Patterning the border ocelli system

One finding of the work presented here was that En/Inv, Dll, and Ci precisely mark domains on the *J. coenia* last-instar forewing disc that correspond to the position and shape of pupal cuticle markings. Much of the pupal cuticle is secreted by the forewing during pupation, and positional associations suggest that the black coloration on the pupal cuticle is produced by the Dll-, En/Inv-, and Ci-expressing wing cells. These findings would indicate that the border ocelli system not only determines eyespot color patterns, but also plays a role in patterning and coloration of the pupal cuticle. Some of these pupal cuticle markings occur where gene expression occurs, but there are no adult eyespots. This suggests that the presence of En/Inv, Dll, Ci, and Spalt in the last-instar wing disc is, by itself, insufficient for eyespot formation. In turn, we speculate that other genes are likely necessary to induce the production of the eyespot focal signal. Alternatively, repressors may be present in some pre-pattern spots expressing En/Inv, Dll, Ci, and Spalt, thus preventing adult eyespot formation, but allowing other patterning elements to form on the cuticle. In any case, our findings support Taira and Otaki [22], who proposed that eyespot foci can function in pupal cuticle patterning. It is important to recognize that gene expression studies such as this one have been extremely helpful in identifying patterning genes that are then subsequently supported by knockdown studies [21, 30–33]. Unfortunately, however, in the *spalt* CRISPR/Cas9 deletion experiments we did not observe effects on pupal markings or anterior white wing spots. We urge caution in overinterpreting negative mosaic results, however. It is possible that because of the variability of *spalt* somatic mutations, that we simply did not generate mutant cells in these regions, that there were pupal viability issues in potentially informative knockouts, or that the mutant Spalt protein may have retained some of its original function. More functional work is required to assess this.

Our work also demands a reassessment of how border ocelli system patterns are determined. From the last-instar Spalt staining (Fig. 3a), we see that seven wing cells have spots of spalt expression, while a subset of five of these cells also show En/Inv, Dll, and Ci co-expression. All this is in spite of the fact that only two of these spots of co-expression will ultimately go on to produce an adult eyespot. Defining the position of the center of the wing cell is a critical step that must precede these gene expression events. Once this position is defined, a combination of different genes can be expressed which ultimately determine whether eyespots, pupal cuticle markings, and/or simple (white) spots ultimately form on the wing. Thus, our results lead us to envision an expanded model of border ocelli system where pattern elements along the anterior–posterior axis are positioned through a shared process, likely involving Spalt, then combinatorial effects of other ligands and transcription factors determine the final characteristic of specific individual elements, i.e., inductive eyespot foci, cuticle markings, simple spots, etc.

### The role of spalt in post-morphogen color pattern specification

The transcription factor Spalt appears to play multiple functions during butterfly wing patterning. For example, functional knockouts show that it plays distinct roles in both vein determination and eyespot patterning [21]. Here we describe an additional role of Spalt in wing margin color patterning. We observed that Spalt is expressed in a discrete line of cells along the proximal boundary of the border lacuna in last-instar wing discs. These are the cells that will become the margin of the adult wing. This expression domain expands during the early pupal stage, after the morphogen induction phase, to encompass a larger domain of scale-building cells along the wing margin. The interface between Spalt-positive and Spalt-negative scale-building cells has the distinctive, species-specific shape as the EIII submarginal bands in both *J. coenia* and *B. anynana* (Fig. 5), suggesting a connection between spalt and marginal band patterning. In *Drosophila*, *spalt* is involved in positioning the wing veins [34]. In the fly wing the transcription factor Knirps is expressed in, and defines, the L2 wing vein, and the positioning of Knirps is controlled by Spalt and Optix [26]. Ultimately the L2 wing vein in *Drosophila* forms at the anterior-edge of the interface or boundary between Spalt-expressing and non-expressing cells [27, 34]. This mechanism has a striking similarity to the proposed positioning of the butterfly EIII submarginal band which appears to be patterned at the boundary between Spalt-positive and Spalt-negative cells in the pupal wing disc (Fig. 5), and in the future it would be interesting to examine Knirps expression to test whether this boundary formation system may have been co-opted for butterfly color patterning.

To test the function of *spalt* in wing margin color patterning, we looked at CRISPR/Cas9-generated *spalt* deletion mosaics in *J. coenia*. Using this technique, previous work demonstrated the importance of *spalt* in eyespot and vein formation [21]. Here, we further show that *spalt* deletion results in loss of submarginal band color patterns on the adult wing (Fig. 6). These results not only confirm that *spalt* is necessary for submarginal band formation, but also suggest it has a highly specialized function in specifically promoting the EIII submarginal band. On both ventral and dorsal wing surfaces we observed mosaics where the EIII element is missing, but the EI and EII marginal bands appear to be undisturbed. This is especially striking in the individuals shown Fig. 6a, where a section of EIII is gone, but the EI and EII bands are unaffected. Furthermore, this loss of EIII also reveals a section of a red background pattern, implying epistasis between *spalt* and this red *optix*-induced element [33]. The epistatic masking of an *optix* color pattern by a Wnt-induced pattern has also recently been shown in *Heliconius* butterflies [32, 35], suggesting a deep conservation of patterning system interactions in which *spalt* appears to play a key role.

The effects of *spalt* knockouts on margin color patterns are quite different than those of *Dll* knockouts, which result in a loss of all wing margin color patterns in both *J. coenia* and *B. anynana* [21]. We infer that, in the context of wing margin color patterning, *Dll* likely plays an early role in determining the entire margin pattern system, consistent with its wing margin expression in last-instar wing discs. Then *spalt* likely plays a later role in specifically elaborating the EIII pattern. Extending this speculative model further, we propose *spalt* as a candidate for a morphogen readout factor in pupal wings, since it has a highly specific role in determining very particular subpatterns of a system likely to be induced by inductive morphogen signaling [18, 32].

## Conclusion

The observations presented here expand our understanding of the role that patterning genes play during butterfly wing development, and further demonstrate the multiple roles that these factors play in color patterning. For example, Spalt not only plays a role in defining eyespot color patterns, but it is also required for wing margin color patterning. These results highlight how evolutionarily novel structures, such as butterfly wing patterns, evolve through the redeployment again and again of conserved, and apparently interconnected, gene regulatory networks.

## Methods

### Antibodies

Rabbit anti-*J. coenia* Dll antibody [8], rabbit anti-Spalt antibodies [36] and mouse cross-reactive 4F11 monoclonal antibody that recognize the Engrailed and Invected proteins have been previously described [8, 37, 38]. Rat anti-Ci antibodies were raised and purified against a glutathione S-transferase fusion protein containing the NH_2_-terminal portion of the *J. coenia* Ci protein.

### Butterfly husbandry

*J. coenia* were originally obtained from Fred Nijhout (Duke University), and *B. anynana* were obtained from the Paul Brakefield (University of Cambridge, UK). *J. coenia* were reared at 28 °C under a 16L:8D photoperiod and fed an artificial diet containing *Plantago lanceolata* [39]. *B. anynana* were raised under a 12L:12D photoperiod and the larvae were fed maize plants.

### Deletion of *spalt* in *J. coenia* using CRISPER/Cas9 genome editing

*spalt* somatic mosaic deletions in *J. coenia* were from an experimental population reported in [21]. Images are available on Dryad: 10.5061/dryad.tj45p.

### Immunohistochemistry

Either last-instar larval or 12–24 h pupal wing discs were fixed for 30 min in 0.1 M PIPES (pH 6.9), 1 mM EGTA, 1% Triton X-100, 2 mM MgSO_4_, and 1.8% formaldehyde. The discs were then incubated in 50 mM Tris (pH 6.8), 150 mM NaCl, 0.5% NP40, and 5 mg/ml bovine serum albumin (BSA) (block buffer) for a minimum of 2 h at 4 °C. The wings were then placed in 50 mM Tris (pH 6.8), 150 mM NaCl, 0.5% NP40, and 1 mg/ml BSA (wash buffer) containing either rabbit anti-Spalt (1:200), or mouse anti-En/Inv (4F11) (1:5)/rat anti-Ci (1:25)/rabbit anti-Dll (1:100) and incubated overnight at 4 °C. The wings were washed 4 times in wash buffer and then incubated for 2 h at 4 °C in wash buffer containing goat anti-mouse FITC (1:200, Jackson Laboratories, West Grove, PA), goat anti-rat Cy3 (1:200, Jackson Laboratories, West Grove, PA), and goat anti-rabbit Cy5 (1:200, Jackson Laboratories, West Grove, PA). The wing discs were washed four times in wash buffer and then placed on glass slides with the Vectashield (Vector Laboratories, Burlingame, CA). Glass coverslips were applied over the discs and images were collected on a MRC600 laser-scanning confocal microscope. Images were individually collected and then assembled using Adobe Photoshop (Adobe Systems Incorporated, San Jose, CA) software.

## Data Availability

Indicated in the text.
